# Cellular and Matrix Organisation of the Human Aortic Valve Interleaflet Triangles

**DOI:** 10.3390/biology14070863

**Published:** 2025-07-16

**Authors:** Najma Latif, Padmini Sarathchandra, Albaraa Al-Holy, Sanida Vaz, Adrian H. Chester, Magdi H. Yacoub

**Affiliations:** 1 Heart Science Centre, Magdi Yacoub Institute, London UB9 6JH, UK; 2 ICTEM Building, National Heart & Lung Institute, Imperial College London, London W12 0NN, UK

**Keywords:** interleaflet triangles, collagen, elastin, fibroblasts, myofibroblasts, smooth muscle cells, myocardium, adipose, nerves

## Abstract

(1) Background: The sophisticated function of the aortic root relies on the coordinated movement of its constituent components. This study examines the extracellular components of the interleaflet triangles (ILTs) and characterises the cells that are present within this region of the aortic root. (2) Methods: A total of 10 human aortic valves and 6 porcine aortic valves were processed for immunohistochemical staining, scanning, and transmission electron microscopy. (3) Results: The three ILTs differed in size and macroscopic appearance. Each triangle comprised up to five distinct layers of tissue: an innermost endothelial layer, an inner elastin-rich layer, a thicker outer layer comprising densely packed layers of collagen and glycosaminoglycans, and an outer layer of intermingled myocardial and adipose tissue. A band of cells near the luminal surfaces of all ILTs expressed smooth muscle cell α-actin with variable expression of smooth muscle myosin heavy chain. In all the ILTs, there was evidence of neurofilament staining, indicating the presence of nerve fibres. (4) Conclusions: Each ILT is unique in its structure and organisation, with differing amounts of elastin and collagen, as well as myocardial, adipose, and fibrous content. The ILTs contain multiple cell types in varying abundance. Functional studies are required to determine the role of the different cells and their organisation in contributing to the sophisticated, dynamic behaviour of the aortic root.

## Simple Summary

The cellular composition and structural components of the valve leaflets, sinus, and annular tissue have all been characterised. In contrast, relatively little is known about the tissue and cellular components of the interleaflet triangles (ILTs). We have used normal human and porcine valves to assess the type of cells present in the different layers and the spatial pattern of the main proteins of the extracellular matrix. We have shown that there are up to five distinct layers of tissue in all the ILTs, and their composition varies between the three ILTs of each aortic valve. This data aids in understanding the functionality of the ILTs and provides essential information when tissue engineering valves.

## 1. Introduction

**Figure 1:**
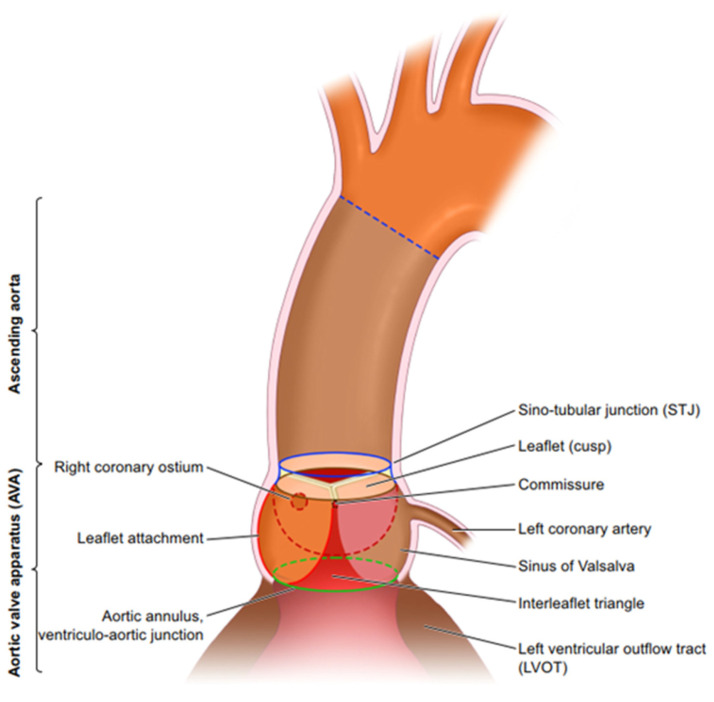
The aortic root includes all the tissue from the beginning of the left ventricle outflow tract in the left ventricle to the sinotubular junction at the beginning of the ascending aorta. Within these boundaries lie the 3 interleaflet triangles, the 3 sinuses of Valsalva, the left and right coronary ostia, the 3 leaflets, and the 3 commissures. From Ref. [5].

The aortic valve is an extremely sophisticated structure that serves to preserve the unidirectional flow of blood from the left ventricle, optimise coronary blood flow, and preserve myocardial function. The complexity of aortic valve function was illustrated in 1999 when it was proposed that the function of the valve relies on “dynamism and crosstalk” [1] (Figure 1). Dynamism is defined by the ability of the component parts of the valve to move spatially and change their size and shape in a coordinated manner [1,2,3,4]. This is possible due to specific mechanical properties of the different components that form the aortic root, which include subvalvular regions, (LVOT, sub-aortic curtain and fibrous trigones), the crown shaped annulus, the leaflets and the supravalvular regions (sinuses of Valsalva and the sinotubular junction) [5] (Figure 1). Between the subvalvular and supravalvular regions are the interleaflet triangles (ILTs) that are interposed between the nadirs of the crown-shaped annulus. Structurally, the ILTs have been described as circularly oriented layers of fibres of collagen covered by an elastic layer that is continuous with the endocardium of the left ventricle [6]. The ILTs are thinner and contain less collagen than the adjacent crown-shaped annulus, to which the leaflets are attached. Two of the ILTs have a myocardial base, while the ILT between the left and non-coronary (L-N) sinus gives way to the sub-aortic curtain. The ILTs are subjected to ventricular haemodynamics [2], and the luminal surfaces experience high shear laminar flow as blood is ejected from the left ventricle and through the aortic valve.

Experience with the Ross procedure has demonstrated the importance of a living valve and the role of cells in maintaining valve integrity and function [7,8]. Consequently, the cells, matrix, and nerve fibres within the valve leaflets, sinus tissue, and the annulus have been characterised [9,10,11,12]. These studies have shown that cells within the leaflets are predominantly a fibroblastic phenotype, with cells having contractile elements, while those in the sinus wall are predominantly smooth muscle cells [12]. The endothelium of valve leaflets has been shown to regulate the stiffness of leaflet tissue [13]. The coordinated movements of different components of the aortic root may be influenced by contractile or dilator stimuli arising from endothelial, fibroblastic, smooth muscle, myocardial, or neuronal mechanisms, as has been demonstrated for valve leaflets [14,15]. All the components of the human aortic root have been extensively studied except for the ILTs. The aim of this study is to characterise the cellular components, the matrix, and their organisation within the ILTs.

## 2. Materials and Methods

Human aortic valves were collected from ten human hearts (age range: 39–67 years; 6 males and 4 females, mean age of 54.85 years, SD of 9.90 years) that were donated to the Royal Brompton Hospital and the John Radcliffe Hospital Valve Banks but were deemed unsuitable for clinical use due to fenestrations in the leaflets. This did not affect their use in this study. This study was approved by the Royal Brompton Hospital ethics review board, and informed consent was obtained from the patients or their relatives. The aortic root was dissected free of surrounding tissue, trimmed just above the sinotubular junction, and opened longitudinally through the centre of one of the sinuses; the cusps were removed, exposing each of the three ILTs. The ILTs were categorised with reference to their anatomical location, namely the triangle between the left and right coronary leaflets (L-R), between the non- and right coronary leaflets (N-R) and between the left and non-coronary leaflets (L-N). Each triangle was then dissected free of the adjacent tissue with a scalpel blade and fixed in 10% formal saline. The central portion with the maximal height of each ILT was dissected out, as well as 2 longitudinal sections from each side of each ILT, all rotated to the sagittal plane and placed inside the tissue cassettes. The tissues were processed, mounted in wax blocks, sectioned, and stained for extracellular matrix and cellular components. The endothelial lining of human ILTs was not completely intact due to handling, so for endothelial studies, we used 6 porcine hearts that were obtained from a local abattoir (Turners’, Farnborough, Surrey), and the ILTs from aortic valves were isolated and fixed in 2.5% glutaraldehyde for scanning electron microscopy and formal saline.

### 2.1. Histological Staining

#### 2.1.1. Elastic Van Gieson Staining for Extracellular Matrix

Dewaxed 5 μm thick paraffin sections were rinsed in 100% methanol, immersed in Miller’s elastin stain (Pioneer Research Chemicals Ltd., Colchester, UK) for 2 h at room temperature, differentiated in 90% methanol for 10–20 s, washed well in tap water followed by distilled water, immersed the slides in Van Gieson stain (Pioneer Research Chemicals Ltd., Colchester, UK) for 5 min, rinsed briefly in distilled water, dehydrated rapidly through graded methanol, cleared in xylene, and mounted in DPX.

#### 2.1.2. Alcian Blue/Sirius Red Staining for Extracellular Matrix

Dewaxed and rehydrated 5 μm thick paraffin sections were stained with Weigert’s Haematoxylin for 10 min followed by washing with tap water and then distilled water. Sections were then incubated with 3% Alcian blue for 20 min followed by 1% molybdophosphoric acid for 20 min. After a brief distilled water wash, the sections were stained with Sirius Red/Picric Acid for 60 min. Stained slides were washed briefly in distilled water, dehydrated rapidly through graded methanol, cleared in xylene, and mounted in DPX.

#### 2.1.3. Immunohistochemistry

The 5 µm thick paraffin wax sections of interleaflet triangles were dewaxed and rehydrated in water. Antigen retrieval was carried out by immersing the slides in 0.1 M citrate buffer (pH 6) and microwaving for 10 min, and the slides were then left in the same buffer for a further 20 min followed by washing with tap water. Endogenous peroxidases in the tissue were blocked by incubating with 3% hydrogen peroxide for 5 min. To reduce non-specific binding, the slides were incubated with 3% bovine serum albumin (BSA-Sigma, St. Louis, MO, USA) + 3% normal horse serum (Vector Laboratories, 2BScientific, Kidlington, UK) for 30 min. The slides were then incubated overnight in a moist chamber with antibodies against CD31 (1/300, Abcam, Cambridge, UK), smooth muscle α-actin (αSMA) (1/3000, DAKO, Cambridge, UK), smooth muscle myosin heavy chain (SMM 1/400, DAKO), von Willebrand factor (1:800 dilution DAKO), cardiac muscle marker troponin I (1:1000 dilution, Abcam), neurofilament protein (1/200, Dako), acetylcholine transferase (1/500, Abcam), tyrosine hydroxylase (1/1000, Genetex, Irvine, CA, USA), and vasoactive intestinal peptide (1/400, Abcam). Negative controls consisted of 3% BSA in PBS. After thorough washing, all the slides were incubated with VECTASTAIN^®^ Elite^®^ ABC-Peroxidase Kit (R.T.U. Universal, Vector Laboratories, 2BScientific, Kidlington, UK) for 30 min for each secondary and tertiary antibody (Vector Laboratories). Specimens were then incubated with DAB (Sigma) for 5 min, washed well in tap water, nuclei were stained with haematoxylin for 1 min, and slides were mounted using Aquatex (VWR). Digital images were taken on a Nanozoomer (Hamamatsu, Shizuoka, Japan). Thickness measurements were acquired using the NDP.view2 software version 2.7.25. A total of 10 regions of thickness from the top to the bottom were measured of the relevant matrix protein along all 3 ILTs from all 10 valves for elastin, collagen, and means were calculated for the 3 ILTs.

### 2.2. Transmission Electron Microscopy (TEM)

1–2 mm cubes of tissue from different depths of the ILTs were fixed in 2.5% glutaraldehyde in 0.1 M sodium cacodylate buffer for at least two hours followed by 2 buffer washes. The specimens were post-fixed with 1% osmium tetroxide in 0.1 M cacodylate buffer for 1 h. After 2 buffer washes, the specimens were dehydrated through an ascending series of acetone starting from 25% to 100%. The specimens were then infiltrated with an acetone/araldite resin mixture (1:1 ratio) overnight in the specimen rotator. After two more resin changes, at least 2 h in each resin, the specimens were placed, noting their orientation, in a rubber mould and polymerised in a 60 °C oven for 18 h. Ultra-thin sections (100 nm) were cut and picked up on copper grids. Post-staining was carried out by floating grids on 20 µL of 2% aqueous uranyl acetate, followed by lead citrate solutions. Grids were viewed in the JEOL 1200 transmission electron microscope (JEOL Ltd., Tokyo, Japan), and digital images were taken using a Gatan Digital Micrograph (Gatan, Pleasanton, CA, USA).

### 2.3. Scanning Electron Microscopy (SEM)

ILTs samples were fixed in 2.5% glutaraldehyde in 0.1 M sodium cacodylate buffer for at least two hours, followed by 2 buffer washes. The specimens were post-fixed with 1% osmium tetroxide in 0.1 M phosphate/cacodylate buffer for 1 h. After 2 buffer washes, specimens were dehydrated through an ascending series of ethanol starting from 25 to 100%. The specimens were air dried using hexamethylene dizilasine (HMDS) and mounted on SEM stubs coated with gold/palladium. Images were taken on a JEOL 6010 analytical scanning microscope (JEOL Ltd., Tokyo, Japan).

### 2.4. Statistics

Data was tested for normality using 3 tests: D’Agostino and Pearson test, Shapiro–Wilk, and Kolmogorov–Smirnov tests. One-way ANOVA was used for multiple comparisons, and a *p* < 0.05 was considered statistically significant.

## 3. Results

The ILTs are not strictly triangles as they have been described in the past [1,6,7] as they are formed by two parabolic sides and a line connecting the adjacent nadirs or fibrous.

### 3.1. Structural Characteristics of the ILTs

**Figure 2:**
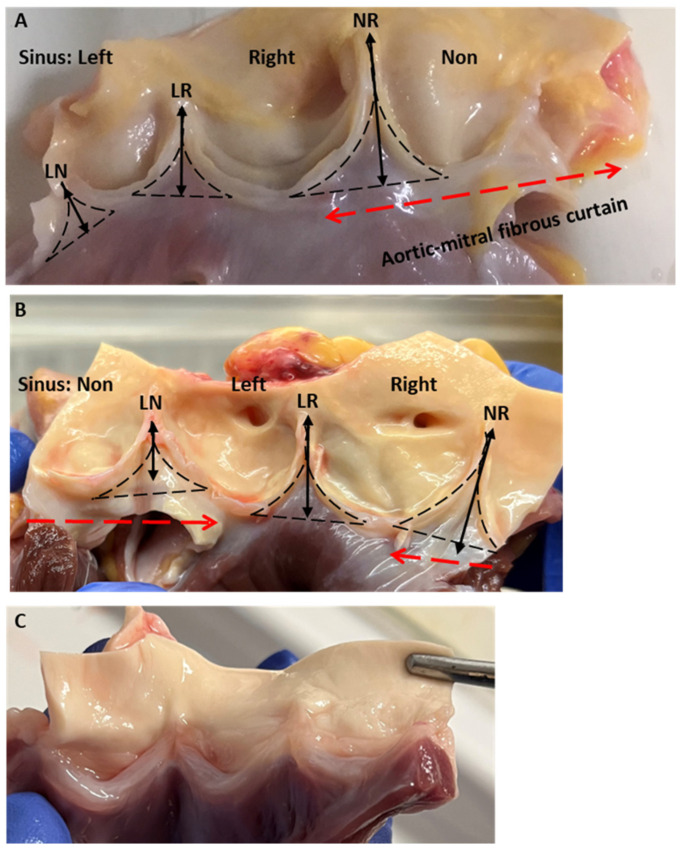
(**A**,**B**) Open view of 2 human aortic valves (AVs) showing the leaflets, the ILTs and the aortic-mitral fibrous curtain (dashed line) and (**C**) a pulmonary valve (PV) for comparison. LN: left to non ILT; LR: left-to-right ILT; and NR: non-to-right ILT.

The three ILTs differed in size and macroscopic appearance, with the N-R triangle being the tallest, while the L-N triangle was the shortest (Figure 2A). Histologically, each triangle comprised five distinct layers and can be described as such from the luminal side: an endothelial lining, an elastin-rich layer, a collagen-rich layer, and myocardial and adipose tissue, with the latter four layers being of different depths. The percentage of the circumference supported by myocardium and fibrous tissue varied between different aortic roots and contrasted with pulmonary valves, where ventricular myocardium is incorporated into the bases of all three of its sinuses and the leaflets are attached to the right ventricular outflow tract myocardium (Figure 2B).

### 3.2. Cellular Components of the ILTs

**Figure 3:**
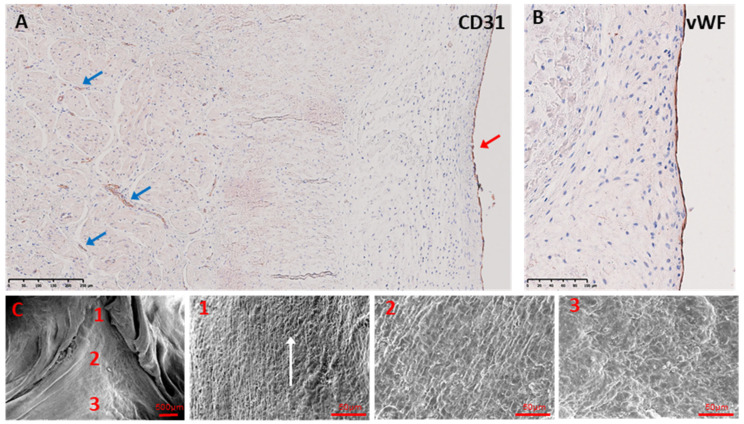
Representative immunoperoxidase staining of CD31 (**A**) showing surface endothelial cells and microvessels in the porcine N-R ILT wall. (**B**) shows vWF (von Willebrand Factor) staining of surface endothelium in porcine N-R ILT wall. Representative SEMs of porcine ILTs (**C**) with magnified zones 1–3. Scale bar is 250 µm in (**A**); 100 µm in (**B**); 500 µm in (**C**), and 50 µm in magnified panels of (**C**). Red arrow in A shows surface endothelial cells, blue arrows show vascular endothelium, and white arrow in C1 shows direction of endothelial cell orientation.

The luminal side of each triangle stained positive for CD31 (Figure 3A) and von Willebrand factor (Figure 3B), indicating a layer of endothelial cells. Endothelial cells of the vessels could be seen deeper in the ILTs (Figure 3A). Scanning electron microscopy (SEM) was performed on porcine ILTs, as the endothelial lining of human ILTs was damaged and not intact. SEM of ILTs from porcine hearts showed that this endothelial covering was arranged in parallel to the direction of blood flow at the apex of the ILTs and became more non-directional towards the bases of the ILTs (Figure 3C).

**Figure 4:**
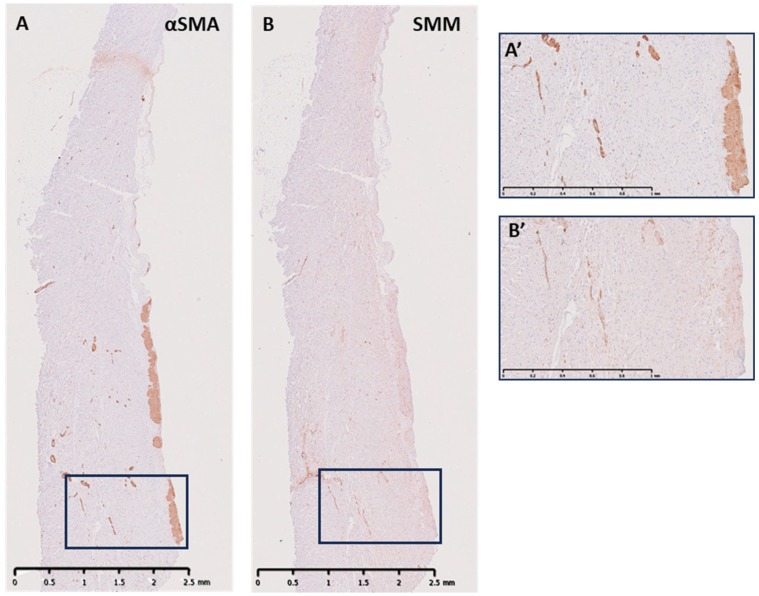
Representative longitudinal sections of human N-R ILT sections showing positive cellular staining for α-smooth muscle actin (**A**) with magnified inset (**A’**) and smooth muscle myosin heavy chain (**B**) with magnified inset (**B’**) showing positive cells running parallel to the surface and to varying heights within the ILTs. Scale bar is 1mm in (A’,B’).

Directly underneath the endothelial layer, there was a band of cells of varied height and depth in the ILTs that was positive for α-SMA (Figure 4A,A’). A proportion of this population of cells expressed the smooth muscle marker smooth muscle myosin heavy chain (SMM-HC), in all three ILTs (Figure 4B,B’). These cells were identified as smooth muscle cells. The cells expressing α-SMA without the expression of SMM-HC were identified as myofibroblasts. Vessels were identified deeper in the ILTs, which stained positive for α-SMA and SMM (Figure 4A,B,A’,B’). The remaining cells within the fibrous layers of the ILTs were identified as fibroblasts using TEM, as there are no specific markers for fibroblasts.

**Figure 5:**
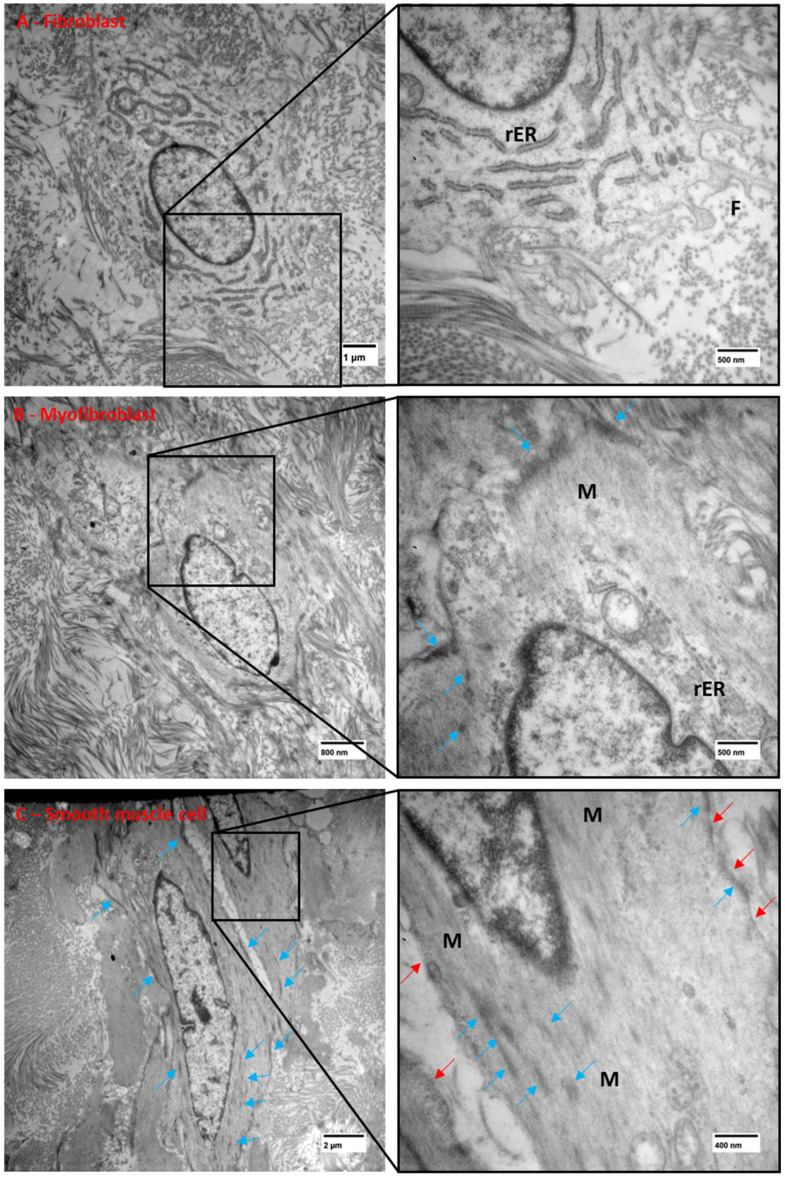
TEM of the different cell types observed in the porcine ILTs: fibroblasts (**A**) showing abundant rough endoplasmic reticulum (rER), cell surface filopodia (F), and an absence of myofilaments and caveolae; myofibroblasts (**B**) showing few rER, some peripheral myofilaments (M), a few focal densities (blue arrows), and few filopodia; smooth muscle cells (**C**) showing very little rER, prominent and abundant surface and cytoplasmic focal densities (blue arrows), caveolae under the cell surface (red arrows), cytoplasm full of myofilaments (M), and no filopodia. Scale bars are 1 μm in (**A**), 800 nm in (**B**), and 2 µm in (**C**).

Key features of all three cell types were confirmed by TEM (Figure 5). The fibroblasts showed no basal lamina or myofilaments, were elongated and spindly with prominent and well-developed rough endoplasmic reticulum, thin filopodia, and occasional small focal adhesions. They were embedded in abundant collagen fibrils (Figure 5A). The myofibroblasts showed a partial basal lamina, few cellular protrusions, the presence of peripheral myofilaments, more and larger focal adhesions, and a well-developed rough endoplasmic reticulum (Figure 5B). The smooth muscle cells were fusiform in shape, showed a complete basal lamina, caveolae under the cell surface, and cytoplasm full of myofilaments, with dense and abundant surface and cytoplasmic electron-dense focal adhesions (Figure 5C).

### 3.3. ECM Elements in the ILTs

**Figure 6:**
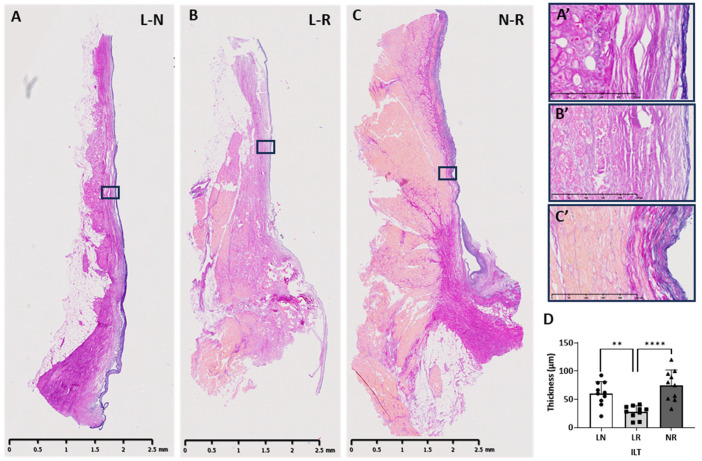
Representative elastic van Gieson (EVG) staining of longitudinally sectioned human ILTs, showing purple coloured elastin fibres running below the luminal surface, magnified images below illustrating elastin fibres, and 2 distinct layers of loosely packed and densely packed collagen fibres (pink): (**A**) L-N, (**B**) L-R, and (**C**) N-R ILTs (scale bar = 2.5 mm) with (**A’**–**C’**) showing respective magnified images (scale bar = 250 µm). (**D**) shows the mean thicknesses of the 3 ILTs. ** *p* < 0.01; **** *p* < 0.001.

The layer underneath the endothelium consisted predominantly of multiple sheets of elastin, which varied in number, running longitudinally and intermingled with collagen. This layer of elastin fibres was highly variable, ranging in thickness from 5.25 to 162 µm (Figure 6). The L-R (mean = 27.90 µm, SD = 21.61 µm) (Figure 6B) and N-R (mean = 72.96 µm, SD = 39.32 µm) (Figure 6C) ILTs contained the thinnest and thickest elastin layers, respectively, and the L-R was significantly thinner than both L-N (*p* = 0.004) (Figure 6A) and N-R ILTs (*p* < 0.0001) (Figure 6D). This elastin layer was distinctive in its structure, being of long, continuous elastin sheets, often thinning towards the apex of each triangle and reducing in abundance with highly variable shorter lengths and thicknesses in the adjacent layers.

**Figure 7:**
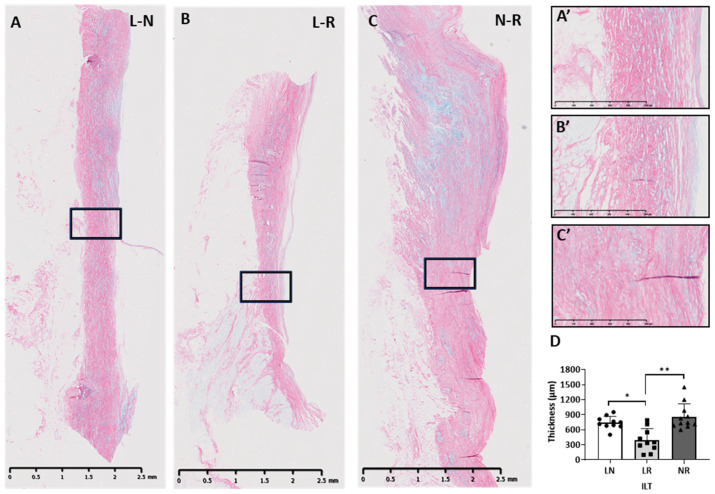
Representative alcian blue and Picrosirius red staining of longitudinally sectioned human ILTs showing the distribution of collagen (red) and proteoglycans (blue): (**A**) L-N, (**B**) L-R, and (**C**) N-R ILTs (scale bar = 2.5 mm) with (**A’**–**C’**) showing respective magnified images (scale bar = 500 µm). (**D**) shows the mean thicknesses of the 3 ILTs. * *p* < 0.05; ** *p* < 0.01.

The third, outer layer comprised predominantly longitudinally arranged collagen fibres with an inner, densely packed and outer, loosely packed collagen layer intermingled with glycosaminoglycans (Figure 7). This densely packed collagen layer was significantly thinner in the L-R (mean = 422.20 µm, SD = 344.90 µm) (Figure 7B) ILT compared to L-N (mean = 664.50 µm, SD = 152.20 µm) (*p* = 0.04) (Figure 7A) and N-R (mean = 719.90 µm, SD = 464.50 µm) ILTs (Figure 7C), (*p* < 0.0089) (Figure 7D). Glycosaminoglycans were abundant within the collagen-rich layer and the elastin layer.

**Figure 8:**
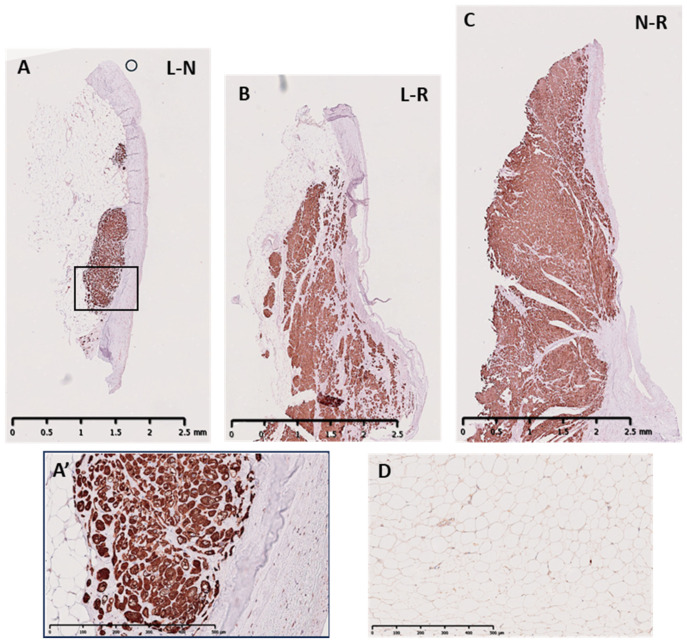
Representative troponin I staining of longitudinal sections of human ILTs showing the extent of myocardial and adipose support: (**A**) L-N, (**B**) L-R, and (**C**) N-R ILTs (scale bar = 2.5 mm) with (**A’**) showing respective magnified image of panel in (**A**) (scale bar = 500 µm). (**D**) shows staining for leptin.

Outside of this collagenous layer was a layer of myocardial tissue, stained using troponin I, which varied along the height and depth of the 3 ILTs (Figure 8). The L-N contained the lowest percentage of myocardial tissue with 33.33% of the samples showing pockets of myocardial tissue along the wall of the ILT (Figure 8A), and 50% of the L-R (Figure 8B) and 66.67% of the N-R (Figure 8C) showed a full myocardial layer from the base to the commissure of the valve. Where the wall of the ILT showed no myocardial tissue, the wall was supported by white adipose tissue, stained using leptin (Figure 8D). As the L-N ILT showed the least amount of myocardial tissue, it had the most amount of adipose tissue. The adipose tissue in all the ILTs was vascularised and innervated.

### 3.4. Neuronal Tissue

**Figure 9:**
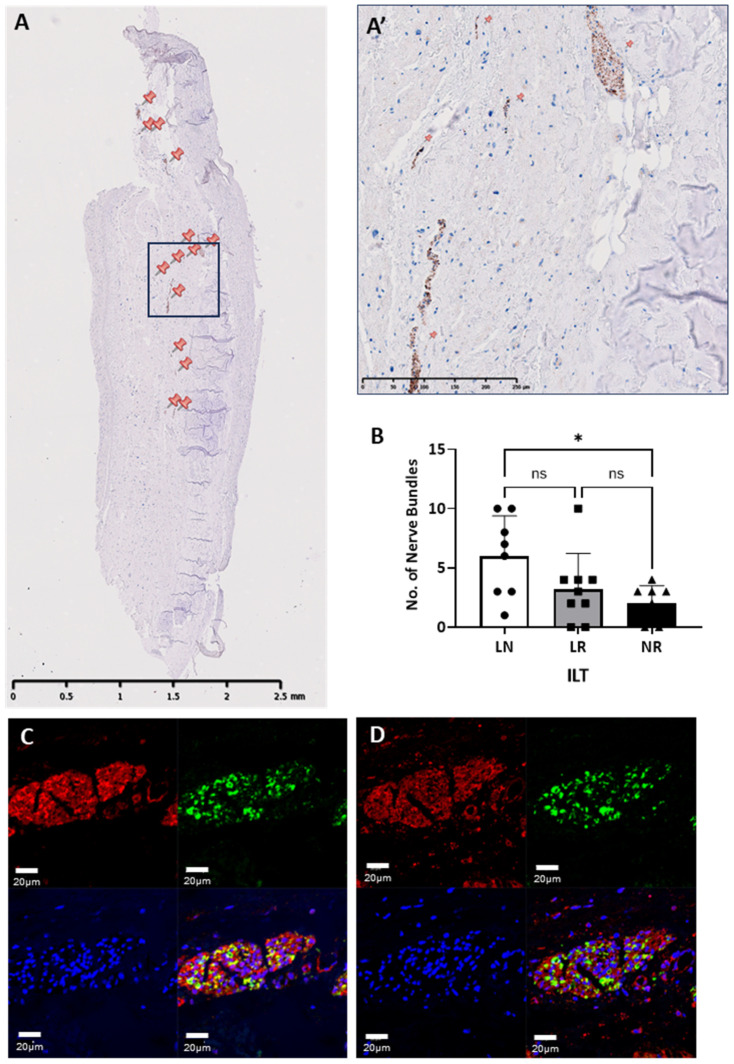
Representative longitudinal sections of an N-L human ILT showing positive neurofilament staining with nerve bundles marked with a pinpoint (**A**) and at higher magnification (**A’**). Graph showing the number of nerve bundles in each ILT (**B**). Colocalisation of acetylcholine (red) and tyrosine hydroxylase (green) in the same nerve bundle (**C**). Colocalisation of VIP (red) and tyrosine hydroxylase (green) in the same nerve bundle (**C**). Scale bars are 2.5 mm in (**A**), 250 µm in (**A’**), and 20 µm in (**C**,**D**). * *p* < 0.05; ns: not significant.

All the ILTs showed the presence of nerve bundles with positive staining for neurofilament protein (NF), and these nerve bundles were usually in the myocardial layer and the adipose tissue (Figure 9A,A’). There was no obvious pattern to the spatial distribution of the nerves in each ILT. However, the incidence of positively stained nerve bundles by NF differed between the ILTs, with the L-N (mean = 6.00 µm, SD = 3.38 µm) and the N-R (mean = 2.00 µm, SD = 1.51 µm) having the highest and lowest number of nerve bundles, respectively. The LN ILT showed a significantly higher number of nerve bundles than the LR (*p* = 0.008) and NR (*p* = 0.004) ILTs (Figure 9B). The majority of nerve bundles expressed the sympathetic marker tyrosine hydroxylase (TH), with fewer nerve bundles expressing parasympathetic markers acetylcholine (ACH) (Figure 9C) and vasoactive intestinal peptide (VIP) (Figure 9D). In some nerve bundles, TH was co-expressed with ACH and with VIP.

## 4. Discussion

This study serves to define the complex composition of the ILTs and discuss the possible role they play in the sophisticated function of the aortic root. This study has confirmed the asymmetry of the human aortic root by analysing the ILTs, and we show heterogeneity in tissue composition between the three ILTs. We show that the N-R triangle was the tallest, while the L-N triangle was the shortest. This observation is in accordance with Izawa et al. [16] and in contrast, in a geometric analysis, two studies found no significant difference in the heights of the three ILTs [17,18]. They also showed that tissue thicknesses in the middle of each ILT were significantly different between all the ILTs [18].

The ILTs comprise five layers: an endothelial layer on the luminal side, a sub-endothelial layer that contains elastin fibres, and a much thicker collagenous layer which comprises densely packed collagen and glycosaminoglycans. We have shown that the N-R ILT has the thickest layer of elastin sheets. This may be a mechanism to manage the increased stresses and strains shown to be present in the non-coronary and right coronary sinuses compared to the left coronary sinus [19]. Clinical series have demonstrated that these sinuses have an increased incidence of dilation or aneurysm compared to the left sinus [20]. The L-R ILT is the smallest and has the thinnest elastin layer.

Regarding the cellular composition, the ILTs have a layer of endothelial cells on the luminal surface. Porcine valves were used only for this aspect of the study, as the endothelial lining covering the complete human ILTs did not remain intact. This is a limitation of the study. The orientation of these cells is parallel to the direction of flow at the apex of the ILTs but more random at the base. The annulus is subjected to different patterns of flow during the cardiac cycle as it changes shape, and these may be responsible for the random orientation of endothelial cells seen at the bases of the ILTs. Under this layer of endothelial cells, there is a population of cells which express contractile proteins, indicating that they are either myofibroblasts or smooth muscle cells.

Studies in mice have shown that the source of cells for the R-L ILT is derived from cells from the neural crest and from the second heart field. In contrast, the tissue for the other two ILTs, the R-N and the L-N, are derived from only the second heart field [21]. Other studies have suggested that the neural crest cells expressing Krox 20 contribute to the formation of the ILT and the hinge region of the leaflets [22]. During development, the ILTs are indistinguishable in composition from the sinus wall and only become evident after birth when the triangular regions interposed between the nadirs of the annulus adapt to form defined structures [21]. This adaptation suggests that the cells within the ILT respond to post-natal changes in blood flow and pressure to specifically remodel these regions of the aortic root.

The precise role of the cells in the ILT is not possible to determine from this histological study. The endothelial cells on the luminal surface of the ILT ensure the continuity of the endothelial coverage of the outflow tract. These cells are exposed to high velocity blood flow since they form part of the left ventricular outflow tract, and as a result, will experience high shear laminar flow, like the endothelial cells on the ventricular surface of the valve leaflets. In a similar manner to the endothelial cells on the valve leaflets [13], these cells may also have the capacity to regulate the stiffness of the ILTs.

Compared to all the other components of the aortic root, the ILTs are the stiffest [23]. The force distribution in the aortic root during the cardiac cycle is not uniform due to the difference in the composition of the component structures of the root, resulting in a change from a circular shape in systole to an oval configuration during diastole [24,25]. The expansion during systole is greatest between the L-R sinus, with the L-R ILT experiencing the greatest radial force, whilst the L-R ILT has the thinnest elastic layer of the three ILTs. This may facilitate the movement of the ILT during the cardiac cycle. The expansion of the aortic root in the subvalvular region allows for the accommodation of a large volume of blood prior to the opening of the valve [3].

Maintaining the structural integrity of the ILT is important to the homeostasis of valve function, allowing for the coordinated movements of each of the aortic root structures. The cells identified in the ILT are predominantly of a fibroblast phenotype, capable of secretion ECM components [23,26]. Without the fibroblastic cells of the ILT, these structures would not have a source of newly synthesised extracellular matrix proteins to maintain their structural integrity.

The presence of contractile cells within each ILT suggests that the size and shape of each triangle can be regulated by biological signals. The ILTs help accommodate the changes in the dimensions of the aortic root during systole. They also assist in the proper coaptation during diastole. The small size of the specimens precluded conducting experiments to measure the contractile capacity of the tissue in response to exogenously added stimulators for the release of endothelial mediators or stimulators of contractile function. These types of experiments have previously shown that porcine leaflets and annular tissue have contractile responses, which were further demonstrated to influence the stiffness of leaflet tissue by up to 25% [13,15,27]. It is possible that the mechanical properties of the ILTs may also have the capacity to be influenced by cellular mechanisms.

The ILTs are richly innervated, and they play a critical role in cardiovascular regulation. We showed nerve fibres expressing acetylcholine transferase, VIP, and tyrosine hydroxylase. The colocalisation of these neurotransmitters has been shown in the heart with distinct stimulation parameters favouring some neurotransmitters over others [28]. The nerve fibres can reduce and increase blood pressure (by activating parasympathetic and sympathetic pathways, respectively), and they may influence aortic valve mechanics, affecting leaflet movement and compliance. Sympathetic activation can increase vascular tone, potentially affecting the flexibility of the aortic sinuses and interleaflet triangles. The sensory nerves detect vessel wall stress, such as dissections, and send pain signals to the brain, often perceived as chest or back pain. Damage to these nerves can lead to significant cardiovascular complications. Procedures like aortic valve replacement (AVR) or root aneurysm repair can potentially disrupt neural pathways, affecting blood pressure regulation and heart rate control.

The interleaflet triangles (ILTs) enhance aortic root flexibility, enabling smooth valve motion, accommodating dynamic shape changes during systole, and ensuring leaflet coaptation during diastole to prevent regurgitation [3]. They help evenly distribute stress across leaflets, maintain laminar blood flow, and reduce turbulence [29]. Biomechanically, ILTs absorb systolic energy via elastic fibres, decreasing leaflet stress, and recoil during diastole to aid valve closure and minimise turbulence through elastic, reversible deformation.

Clinically, ILT dilation is linked to valve dysfunction, aneurysm, dissection, or regurgitation. The Yacoub remodelling technique seeks to preserve or reconstruct ILTs to restore valve function. Abnormal ILT development contributes to congenital and acquired valve disease. A limitation of this study is the small sample size—10 human and 6 porcine aortic roots.

## 5. Conclusions

The ILTs are essential for the structural integrity and function of the aortic root. This study further illustrates the biological complexity of the aortic root and its asymmetry in its macro and microstructure. Understanding the structure and cellular phenotypes of all the components of the aortic root provides insight into the functionality of a normal valve, which should be taken into consideration both in reparative procedures as well as in the development of the next generation of tissue-engineered valves.

## Data Availability

The original contributions presented in this study are included in the article. Further inquiries can be directed to the corresponding author.

## Abbreviations

The following abbreviations are used in this manuscript:

αSMA	Smooth muscle α-actin
ACH	Acetylcholine transferase
BSA	Bovine serum albumin
ILT	Interleaflet triangle
L-N	left-to-non-interleaflet triangle
L-R	left-to-right interleaflet triangle
N-R	non-to-right interleaflet triangle
NF	Neurofilament
SMM	Smooth muscle myosin
TH	Tyrosine hydroxylase
VIP	Vasoactive intestinal peptide
